# Production of hypoallergenic milk from DNA-free beta-lactoglobulin (BLG) gene knockout cow using zinc-finger nucleases mRNA

**DOI:** 10.1038/s41598-018-32024-x

**Published:** 2018-10-18

**Authors:** Zhaolin Sun, Ming Wang, Shiwen Han, Shuangyu Ma, Zhiyuan Zou, Fangrong Ding, Xinrui Li, Ling Li, Bo Tang, Haiping Wang, Ning Li, Huilian Che, Yunping Dai

**Affiliations:** 1 State Key Laboratory for Agrobiotechnology, College of Biological Sciences, China Agricultural University, Beijing, China; 2 Beijing Advanced Innovation Center for Food Nutrition and Human Health, College of Food Science & Nutritional Engineering, China Agricultural University, Beijing, China; 3 Beijing Genprotein Biotechnology Company, Beijing, China

## Abstract

The whey protein β-lactoglobulin (BLG) is a major milk allergen which is absent in human milk. Here, we for the first time generated DNA-free *BLG* bi-allelic knockout cow by zinc-finger nuclease (ZFNs) mRNA and produced BLG-free milk. According to the allergenicity evaluation of BLG-free milk, we found it can trigger lower allergic reaction of Balb/c mice including the rectal temperature drop and the allergen-specific immunoglobulin IgE production; BLG free-milk was easily digested by pepsin at 2 min, while BLG in control milk was still not completely digested after 60 min, and the binding of IgE from cow’s milk allergy (CMA) patients to BLG free-milk was significantly lower than that to the control milk. Meanwhile, the genome sequencing revealed that our animal is free of off-target events. Importantly, editing animal genomes without introducing foreign DNA into cells may alleviate regulatory concerns related to foods produced by genome edited animals. Finally, the ZFNs-mediated targeting in cow could be transmitted through the germline by breeding. These findings will open up unlimited possibilities of modifying milk composition to make it more suitable for human health and also improve the functional properties of milk.

## Introduction

Cow’s milk is a food ingredient that is consumed globally because of its rich nutrients such as protein, fat, carbohydrate, and mineral contents. However, cow’s milk is also one of the most common foods which usually cause allergic reactions^1^. Cow’s milk allergy (CMA) is a common disease in infancy and childhood, and its prevalence approximates 0.3%~3.5% along with rising trend^2^, which severely affect absorption and utilization of nutrients in dairy products^3^.

Although breeding strategies, nutritional management, and quantitative genetics have improved milk yield, these approaches have not led to significant changes in milk composition^4^. With the development of biotechnology, especially in livestock, there will be great opportunities to generate new value-added products for designing milk for human health benefits^5^. Various transgenic cows have been reported for different applications, such as the expression of pharmaceutical proteins^6–9^, increased milk casein protein^10^, improved resistance to disease^11–13^, and improved animal welfare^14,15^. However, an effective method to reduce the allergic reaction induced by milk has not been developed. Bovine milk contains a variety of allergen proteins such as casein, β-lactoglobulin (BLG) and α-lactalbumin, with the milk whey protein BLG as a major milk allergen^16^. Cow’s milk protein allergy affects up to 2-3% of newborns, and the trend is increasing^17^. Different methods have been used to diminish the allergenicity of the BLG, including heating, high pressure, enzymatic hydrolysis and glycation^18,19^. Although these methods can reduce the BLG allergenicity to a certain extent, the structure and function of other proteins in cow’s milk has been damaged which greatly influence the nutritional functions of milk, and the sensitization of BLG is not completely eliminated. By contrast, knocking out the *BLG* gene by gene-editing technology is a more direct approach to completely solve the problem, which is of great significance to the research of hypoallergenic dairy product.

We previously produced a live cloned cow with the *BLG* gene bi-allelic modified via ZFNs, but the gene contained small in-frame deletions and did not create functional *BLG* KO alleles^20^. Recently, BLG-free and high-casein milk was successfully produced from the *BLG* knockdown transgenic cow which was generated by miRNA^21^. This pioneer research firstly proved that the BLG protein in milk can be removed by transgenic methods. However, this method required a puromycin selection cassette and yielded many transgenic copies. And so far, the evaluation of the allergenicity of the BLG-free milk has not yet been investigated. For evaluating the allergenic potential of food proteins, animal models are a useful tool for a direct assessment of the sensitizing potential of food^22^, such as the Balb/c mice model^23–25^. One of the important characteristics responsible for the allergenicity of food proteins is their stability against pepsin digestion^26^. In an *in vivo* study, a pepsin-resistant protein increased the intestinal permeability by 15-fold^27^, thereby enhancing the chance of the sensitization of animals to the exposed protein. Human serum studies may assess the clinical reactivity of food allergens based on the specific IgE levels^28–30^. All these methods can be used to synthetically assess the allergenicity of the BLG-free milk.

Recently, new programmable nucleases transcription activator-like effector nucleases (TALENs) and RNA-guided endonucleases (RGENs) have been used for genome editing in livestock^31,32^. However, mutated farm animals which contained the foreign programmable nucleases DAN or selective marker-gene might be considered genetically modified organisms (GMOs) by regulatory authorities in certain countries, which could reduce the potentially widespread use of programmable nucleases in farm animal biotechnology and agriculture. So, it is important for development of the DNA-free genome editing technology to alleviate regulatory concerns related to genetically modified animals.

In this study, we generated a normal live DNA-free *BLG* bi-allelic knockout cow by ZFNs mRNA. Meanwhile, the natural expressed milk from the *BLG* bi-allelic knockout cow is BLG-free and hypoallergenic. Moreover, the genome sequencing revealed that our animal is free of off-target events and the modifications were stably transmitted to the progeny by multiple ovulation and embryo transfer (MOET)^33^. This finding provides a solid foundation for “designer milk” in future.

## Results

### Generation of the DNA-free *BLG* bi-allelic mutations cow

**Figure 1:**
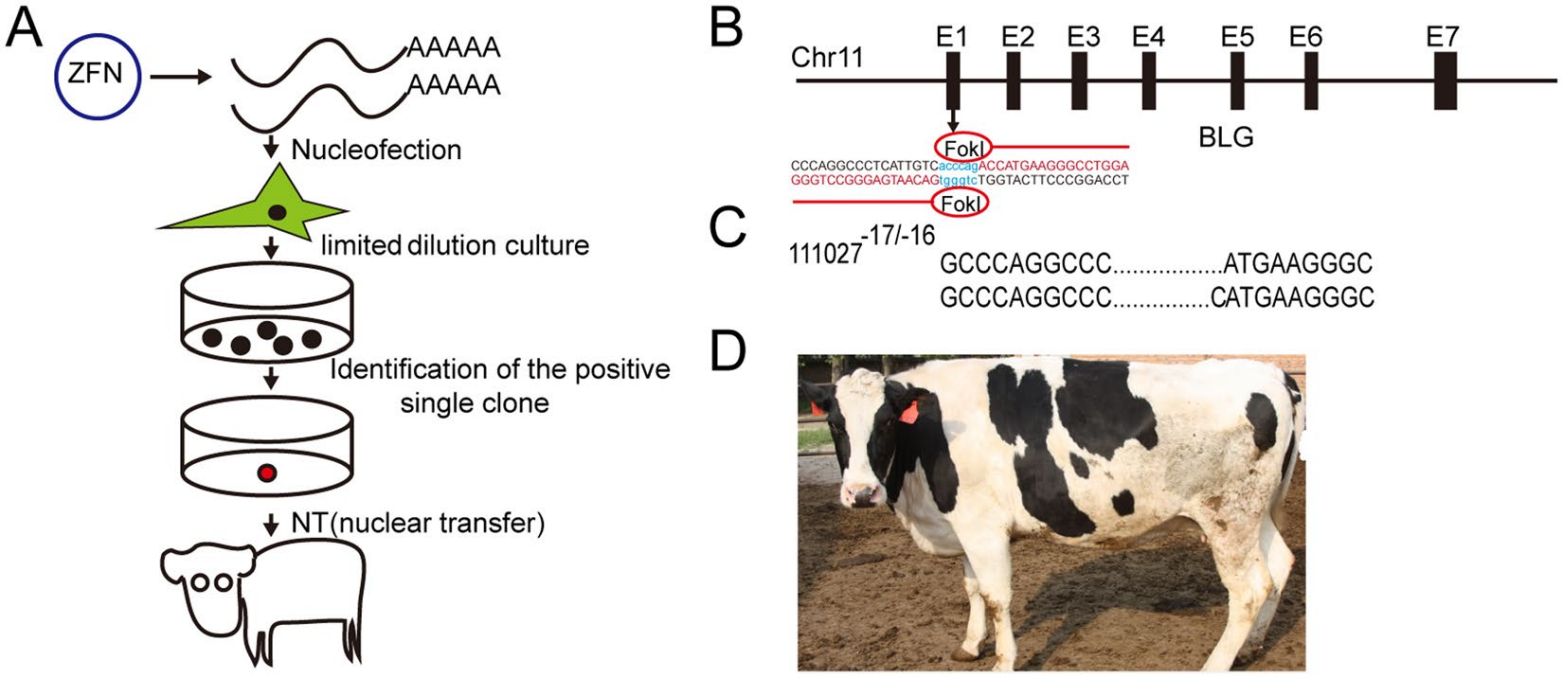
Generation and identification of the DNA-free *BLG* bi-allelic knockout cow. (**A**) Flowchart depicting the methodology used to generate DNA-free gene targeting cow by ZFNs mRNA and SCNT. The mRNA was transcribed from the BLG-ZFNs plasmid *in vitro*, and the BFF cell line was derived from a 46-day-old Holstein cow fetus. The BFF cells were transfected with BLG-ZFNs mRNA using Neocleofector. After 24–48 h, limiting dilution was used to form single-cell colonies at a cell concentration of approximately 500 cells/dish (10 cm^2^). The single-cell colonies were generated after culturing for an additional 7 to 10 days. The positive single-cell colonies were identified by sequencing and as donor to perform NT to generate the DNA-free *BLG* bi-allelic knockout cattle. (**B**) Section of the *BLG* gene targeted by ZFNs. The region targeted by ZFNs was in E1. The DNA sequence of the primary binding site for each ZFNs is colored red. The cut sites cleaved by dimerization of the FokI nuclease domains are blue. (**C**) Identification of the ZFNs-targeted cow. Sequence analysis revealed that the cloned cow was a bi-allelic mutant at the *BLG* locus (−17 bp/−16 bp). (**D**) Photograph of the bi-allelic mutant cow #111027 at 10 months of age.

Previously, we generated one live bi-allelic mutation cow by the ZFNs mRNA, but this cow had small in-frame deletions and did not have the function *BLG* KO alleles required for the production of BLG-free milk^20^. Therefore, we re-screened cells without selective-marker and chose the frame-shift mutations that led to the production of null alleles by the ZFNs mRNA, which as the donor to perform nuclear transfer (NT) to generate the DNA-free *BLG* bi-allelic mutations cow (Fig. 1A). The ZFNs recognized a 40 bp sequence that flanked the 6 bp cleavage site, in Exon 1 of the *BLG* gene (Fig. 1B). For the *BLG* gene disruption, 128 single cell colonies were obtained and sequenced, the clone #112 had bi-allelic mutations, including −17 bp and −16 bp indels that could lead to frame-shift mutations (Table 1). Using this cloned cell as nuclear donors for somatic cell cloning, 2 cloned animals were born. One died soon after birth due to the commonly observed effects of somatic cell cloning (Table 2). Sequencing revealed that the remaining live animal #111027 had a bi-allelic mutation (−17 bp/−16 bp) (Fig. 1C), and the animal is healthy (Fig. 1D).

**Table 1:** Cell screening of ZFNs transfection.

Cell line	Culture method	Isolated clonies	Bi-allelic frame-shift mutation	Freezing cell clonied	Cell clonied for NT
0904FFB	Single cell culture	128	1(−16 bp/−17 bp)	1	#112

**Table 2:** Summary of nuclear transfer results.

Cell clone	Oocytes	Reconstruct embryo	Blastocysts	Blastocysts rates%	Recipients	Pregency day at 60	Live cows
0904FFB #112	321	182	64	35.2	16	5	1

### Assessment of the BLG in the milk from the *BLG* bi-allelic mutations cow

**Figure 2:**
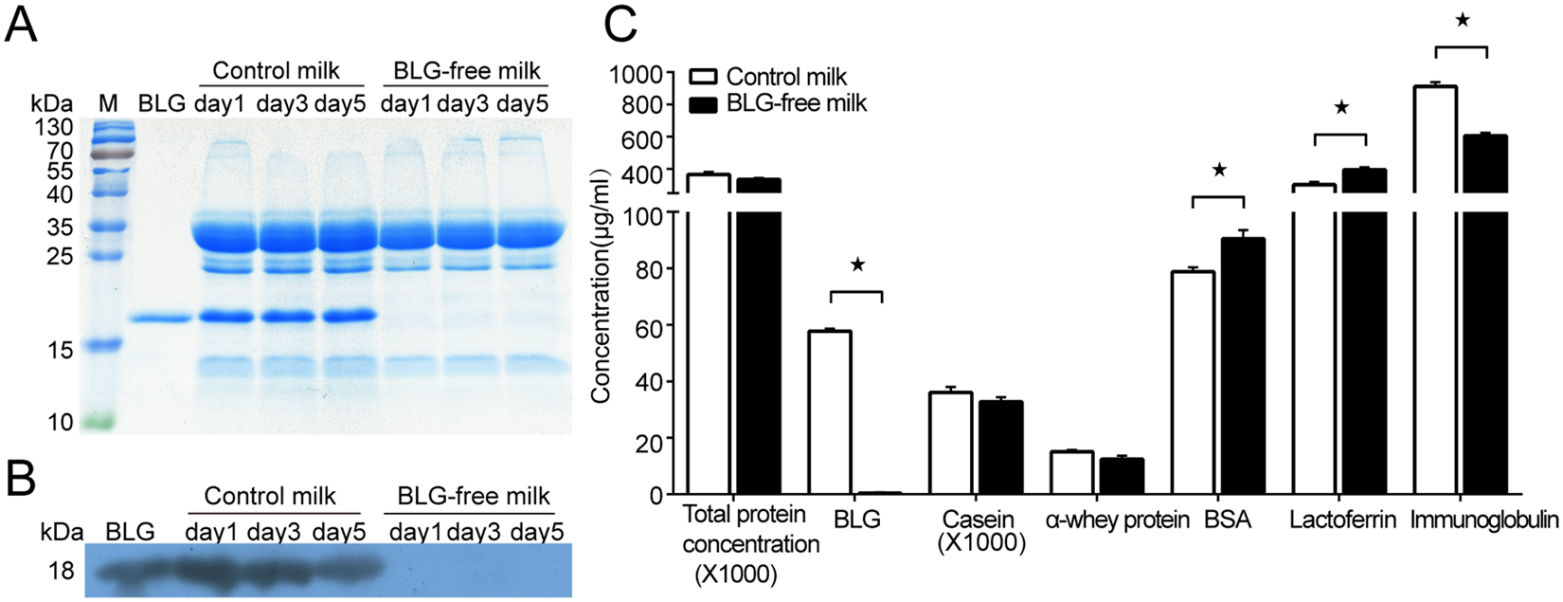
Identification of the expression of the BLG in the milk. (**A**) Characterization of the BLG-free milk by SDS-PAGE. M, protein marker; BLG, 2.5 µg of commercial BLG (Sigma) as a positive control; line 3–5, 1 µl of wild-type cow raw milk from different days; line 6–8,1 µl of #111027 (BLG-free) raw milk from different days. (**B**) Characterization of the BLG-free milk by Western blot. BLG, 2.5 µg of commercial BLG (Sigma) as a positive control; line 2–4, 1 µl of wild-type cow raw milk from different days; line 5-7, 1 µl of #111027 (BLG-free) cow raw milk from different days. (**C**) Concentration of the main proteins in the control milk and BLG-free milk. Results are shown as means ± SD. ^*^P < 0.05, significantly different from control milk.

To confirm the knockout of BLG expression, we obtained natural expressed milk on different days from the *BLG* bi-allelic cow #111027. Analysis of the milk samples by SDS-PAGE and Coomassie blue staining revealed that none of the milk samples from the #111027 cow contained detectable levels of BLG. In contrast, BLG was readily detectable as a major milk protein in wild-type (WT) control milk (18 kDa, Fig. 2A). A more sensitive analysis of BLG levels by Western blotting confirmed the knockout of BLG expression because all milk samples from the gene-editing cow were devoid of any detectable BLG (18 kDa, Fig. 2B). We also detected the concentrations of main proteins in milk using enzyme-linked immune sorbent assay (ELISA). Although BLG was absent in the milk of #111027 cow, the total protein was not altered because of the improvement of expression of other endogenous proteins, such as bovine serum albumin (BSA) and lactoferrin (Fig. 2C). All these results indicated we generated a normal live DNA-free *BLG* bi-allelic knockout cow #111027 by ZFNs mRNA, and the natural expressed milk from #111027 is BLG-free.

### Evaluation of BLG-free milk allergenicity by Balb/c mice food allergy model

**Figure 3:**
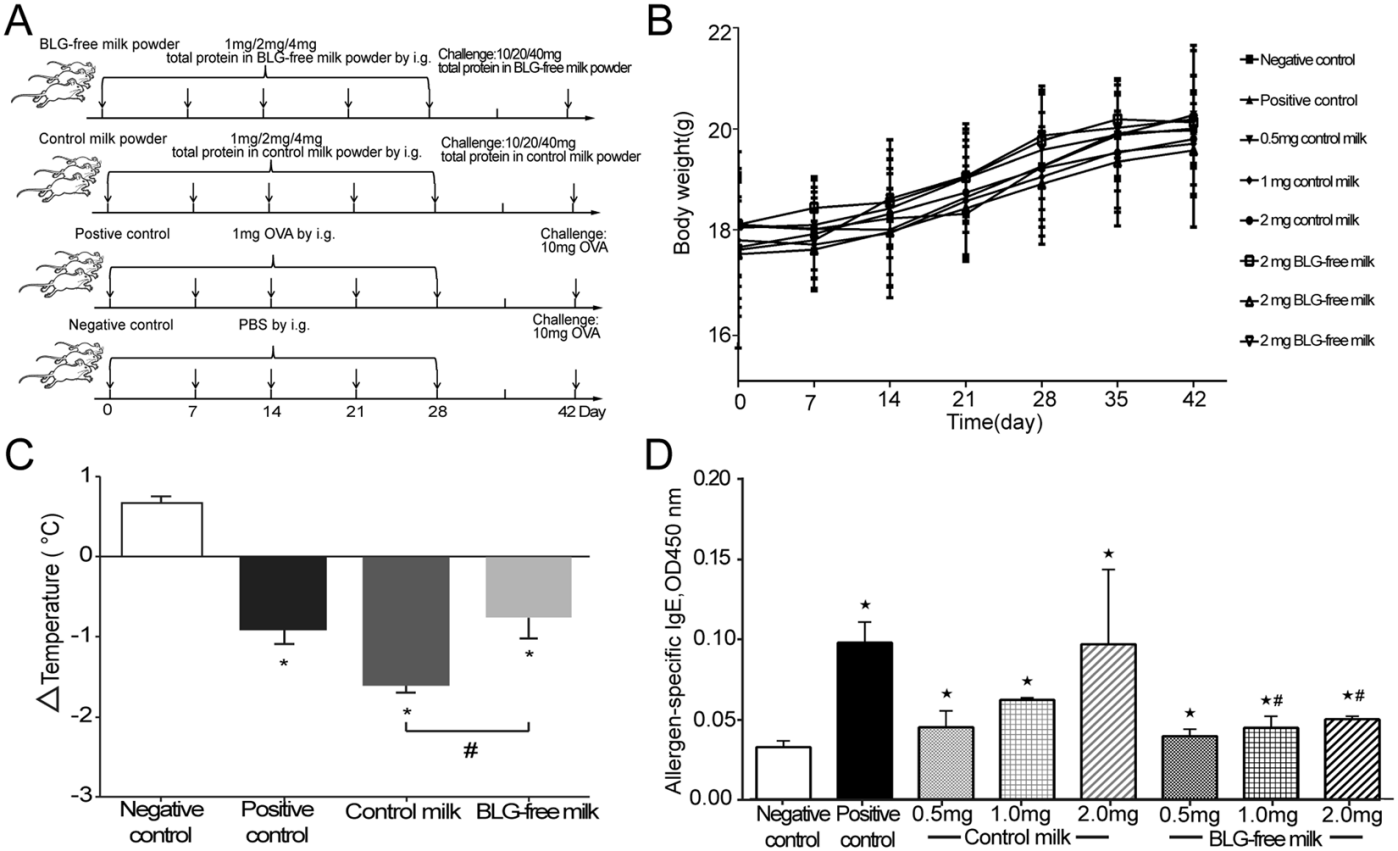
Analysis of the Balb/c mice Food allergy model. (**A**) Schematic drawing representing the Balb/c mice food allergy model protocols and doses used in this work. (**B**) Mean weekly body weight of mice. (**C**) The variation of rectal temperature (°C). (**D**) The level of allergen-specific IgE in serum. Results are shown as means ± SD. ^*^P < 0.05, significantly different from the negative control and ^#^*P* < 0.05, significantly different from the control milk.

To evaluate the allergenicity and nutrition of BLG-free milk, we established the food allergy model in Balb/c mice as shown in Fig. 3A. We weighted and recorded weekly the body weight to assess the nutrition in Balb/c mice, and we found there was no difference between the control milk and the BLG-free milk (Fig. 3B). To assess the allergenicity of BLG-free milk, we firstly detected the rectal temperature, which can reflect the degree of food allergy, of sensitized mice before and after a challenge for 40 min. Ovalbumin (OVA), a positive allergen as a positive control, control milk and BLG-free milk all can lead to the rectal temperature drop of Balb/c mice, however, in the BLG-free group it was significantly decreased compared to the control milk group (Fig. 3C), which can indicate BLG-free milk can trigger a lower allergic reaction than the control milk. Then we detected the allergen-specific IgE level in serum of all tested mice. Compared to the negative control, OVA, control milk and BLG-free milk all can increase the allergen-specific IgE level, however, BLG-free milk has a significant lower allergen-specific IgE production compared to the same concentration of control milk (medium and high group, Fig. 3D). From the results of food allergy model in Balb/c mice, we reached a conclusion that BLG-free milk can decrease the rectal temperature drop and the allergen-specific IgE production, which indicated that it has a lower allergenicity.

### Evaluation of BLG-free milk allergenicity by pepsin digestion assay and human serum IgE binding analysis

**Figure 4:**
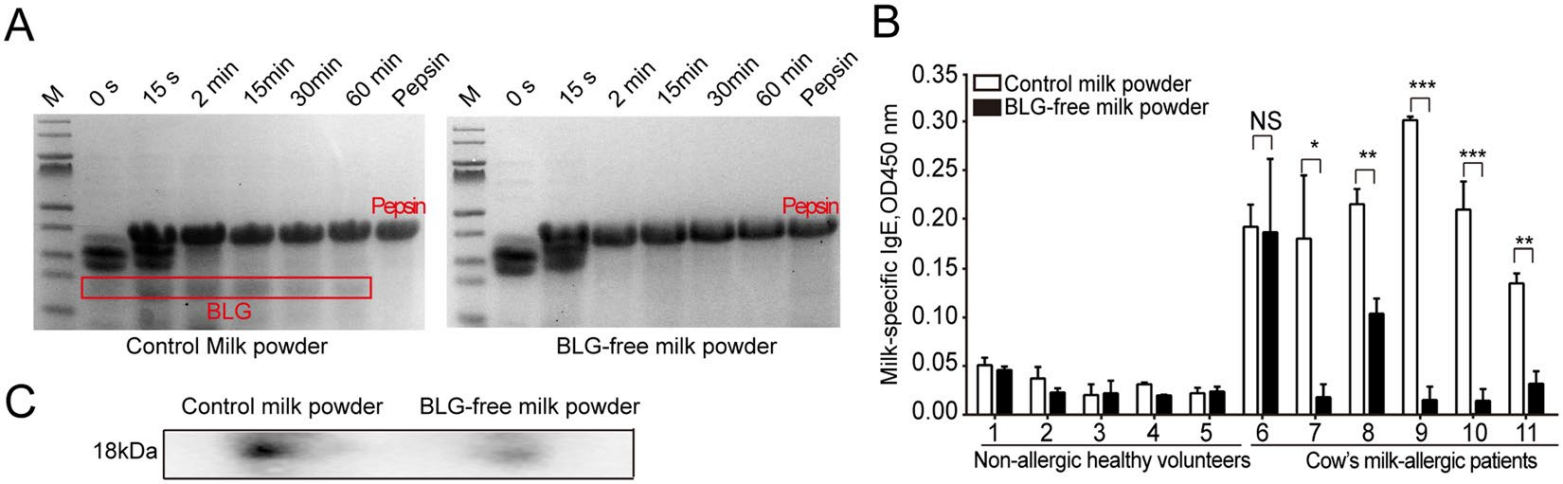
Pepsin digestion assay and serum IgE binding analysis. (**A**) Pepsin digestion assay. Control milk power (left) and BLG-free milk powder (right). Lane 1: molecular weight markers; lanes 2-6: samples of digests at 0 s, 15 s, 2 min, 30 min and 60 min; lane 7: pepsin control. (**B**) IgE-binding capacities of different milk powders to sera from non-allergic volunteers and CMA patients. 1-5: different non-allergic volunteers; 6–11: different CMA patients; white box: Control milk power; black box: BLG-free milk powder. (**C**) IgE reactivities of different milk powders shown with immunoblotting. Lane 1-2: Control and BLG-free milk powders. Results are shown as means ± SD. ^*^P < 0.05; ^**^P < 0.01; ^***^P < 0.001; NS, not significant.

If a natural protein had to maintain its allergenicity, the amino acid fragments of the IgE-binding sites of the protein would generally tolerate pepsin digestion. Thus, these proteins could be absorbed by intestinal mucosa and induce an immune response^34^. Therefore, the protein fragments usually showed stronger allergenicity, which could still be detected after 60 min of *in vitro* digestion. In the pepsin digestion results, 5 mg/mL milk powder samples were taken at 0 s, 15 s, 2 min, 30 min and 60 min and were examined by SDS-PAGE. The BLG-free milk powder was easily digested by pepsin at 2 min, while BLG (18 kDa) in the control milk powder was still not digested after 60 min of treatment (Fig. 4A). Meanwhile, serum IgE binding analysis was also performed to detect the binding capacity of BLG in two kinds of milk and specific-IgE in CMA patients’ serum. We detected the binding capabilities of specific sera from five non-allergic healthy volunteers (serum number 1-5) and six CMA patients allergic to cow’s milk (serum number 6-11) to the control milk and the BLG-free milk using two different methods: ELISA and Western blotting. From the ELISA results (Fig. 4B), the binding capabilities of the BLG-free milk powder to serum number 7-11 from CMA patients were significantly reduced compared to those of the control milk powder (^*^P < 0.05, ^**^P < 0.01, ^***^P < 0.001). Additionally, there was nearly no binding between the sera of non-allergic healthy volunteers and cow’s milk. Notably, the binding of the serum number 6 from a CMA patient was unaffected by the BLG-free milk, probably because the milk contains a variety of allergens and BLG was not the relevant allergen for this patient. Similarly, BLG was the major allergen for serum number 9 and 10 with extremely significant differences (^***^P < 0.001); it was also one of allergens for serum number 7, 8 and 11 with varying degrees of differences (serum number 7 and 11, ^*^P < 0.05; serum number 8, ^**^P < 0.01). Then, we further confirmed whether the decreased serum binding levels were also induced with a mixed pool of CMA patients allergic to cow’s milk (serum number 9 and 10) using Western blotting. In the control milk, there was an apparent band at 18 kDa, the molecular weight of BLG, compared to the BLG free-milk (Fig. 4C). With the combination of the pepsin resistance and serum IgE binding analysis, we reached the conclusion that BLG free-milk produced from *BLG* knockout cow had lower allergenicity, which could be used to relieve the suffering of patients who are allergic to cow’s milk.

### Off-target analysis of the *BLG* bi-allelic mutations cow

Off-target effect is a major drawback concern of the ZFNs. To evaluate off-target effects in the DNA-free *BLG* bi-allelic knockout cow that whether there were any insertion-deletions (indels) ascribable to off-target DNA cleavage by the ZFNs. We sequenced the genome of the mutant cloned cow #111027 to an average of 19 × coverage using the Illumina HiSeq platform (PE125). After mapping the sequenced reads onto the bovine reference genome (UMD_3.1.1(UCSC)), we identified the ZFNs-specific indels. We assessed all remaining potential indels for their proximity to degenerate ZFNs off-target sites, allowing degeneracy of up to 10 bp mismatches per ZFNs pair, 5 bp for each ZFNs monomer. We also allowed spacer length to vary from 4 bp to 16 bp, instead of the optimum of 6 bp contained in the on-target site. This degeneracy expanded our considered target space from one site (*BLG*) of 40 bp in length, to 497 sites (Table 3). Only our intended editing mapped to within 20 bp of any of the identified degenerate targets. These results revealed that our animals are free of off-target events and further supporting the high specificity of ZFNs mRNA, particularly for this locus.

**Table 3:** Degenerate ZFN sites in the bovine genome.

^#^Degeneracy in A TALEN pair	^#^Off-target Genome wide	^#^De novo indels
		111027
		Within 20 bp of indel
0_0^*^	1	1
1_4	0	0
1_5	0	0
2_4	1	0
3_3	0	0
2_5	0	0
3_4	3	0
3_5	19	0
4_4	7	0
4_5	112	0
5_5	354	0
total	497	1

^*^On-Target site.

### *BLG* mutation can be stably passed to the next generations by germline transmission

**Figure 5:**
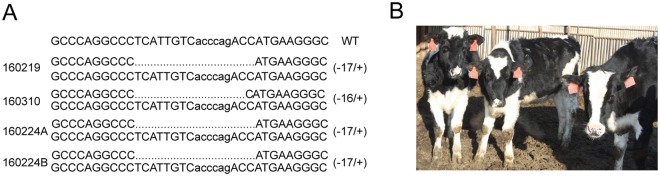
Germline transmission of *BLG*-targeted modification. (**A**) Identification of the F_1_ *BLG* knockout cows by sequencing. (**B**) The image of the F_1_ cows.

The germline transmission of transgenic mutations is an important concern. Therefore, we also investigated whether the targeted allele of the *BLG* gene could be transmitted through the germline. At ~16 months, the MOET method was used to multiply the progeny of the female *BLG*^−/−^ founder #111027. Four F_1_ offspring were born, and the genotypes were monitored via PCR. As expected, all the progeny inherited one mutant allele from their genetically altered parent (Fig. 5A). Currently, the F_1_ offspring were normal live (Fig. 5B).

## Discussion

In this study, we produced a DNA-free B*LG* biallelic knockout cloned cow by SCNT using ZFNs mRNA-targeted cells. The milk from the cloned cow was BLG-free and hypoallergenic. BLG belongs to the strong allergic lipocalin family, and breast milk does not contain BLG^35^. In cow’s milk, BLG presents in the form of biopolymer with molecular weight at 36 kDa, accounting for about 50% of whey protein and about 10% of total milk protein. BLG is a highly-structured protein and can resist acid and protease hydrolysis. After gastric intestinal digestion, it still retains the integrity of the protein or the sensitive fragments, which can cause allergic reactions. Studies have indicated that about 60% of CMA mediated by IgE is caused by BLG^36^, therefore, BLG is considered to be one of the most important allergens in milk. So, the production of *BLG* knockout cow is important to improve the quality of the milk and make it more suitable for cow’s milk allergic patients.

It has been reported that the *BLG* knockdown cattle has been generated by the miRNA^21^. Although the induced milk of this cattle was BLG-free, the natural lactation milk was not detected. Meanwhile, this method required antibiotic resistance genes and yielded many transgenic copies; moreover, the only one live transgenic calf did not have a tail. It is well known that the existence of foreign marker genes interferes with the expression of neighboring endogenous genes^37^, hampers phenotypic and genetic analyses^38^, meanwhile, it may also provoke public concerns regarding biological safety. Thus, it was important to generate the marker-free genome-modification livestock. The marker-free genome-modification pigs, sheep and cattle embryo have been generated by zygote injection of CRISPR/Cas system^39,40^, but this method mostly resulted in mosaicism of the modification and many random mutations. One or two rounds of further breeding should be performed to obtain homozygotes with identical genotype and phenotype. Recently, zygote-mediated DNA oligonucleotides (ODNs) knock in has been reported in cattle which may alleviate some defects^41^. To fully address this issue, a new method has been reported that gene-targeted somatic cells can be used as donor for somatic cell nuclear transfer (SCNT) to produce gene-targeted pigs with single and identical mutations. However, these reports also used the antibiotic resistance genes^31,32,42^ and contained the constitutive expression of CRISPR/Cas9 components in the gene-targeted clones^32^, which might also lead to increased frequencies of off-target mutations^43–46^.

Recently, marker-free, nonmeiotic allele introgression gene-editing pigs and cows has been generated by using TALENs mRNA^14,15^. In the present study, using the ZFNs mRNA, we also generated the normal live DNA-free *BLG* biallelic knockout cloned cow without any other gene-components and the milk was BLG-free, which paly an important role on the acceptance of edited livestock for human consumption in future. Moreover, it has been recently reported that a chemically defined maturation medium supplemented with three cytokines (FGF2, LIF, and IGF1) in combination, so-called “FLI medium”, improves quadrupling efficiency in production of genetically modified pigs by SCNT^47^. With the development of SCNT and the more efficient, convenient CRISPR/Cas9 technology^48,49^, these may accelerate the production of the marker-free gene-editing livestock in future.

Although it has been generated the BLG-free induced milk by transgenic method^21^, so far the allergenicity of BLG-free milk has not been investigated. Thus, in this study we evaluated the allergenicity of BLG-free natural expressed milk using the Balb/c mice food allergy model, pepsin digestion assay and human serum IgE binding analysis. We found that BLG-free milk can trigger a lower allergic reaction and has the same nutritional value compared to the control milk. The rectal temperature declined, and the milk-specific IgE level of tested Balb/c mice in the BLG-free milk group decreased significantly compared with that of the control milk group. Meanwhile, there was no significantly difference in the body weight between the two groups. Then, the pepsin digestion assay and human serum IgE binding analysis proved again its hypoallergenicity. BLG-free milk is easier to be digested by pepsin. We also detected its binding capacity to the serum of CMA patients. In some of the CMA patients’ serum, the bindings were significantly decreased (serum number 7-11 from CMA patients), while there was no change in serum number 6 from a CMA patient because BLG is not the major allergen of this patient. Our BLG-free cow produced high-BSA and lactoferrin milk. Importantly, bovine lactoferrin is a multifunctional glycoprotein involved in intestinal iron absorption and antimicrobial activity^50,51^, which has also been added to milk in yogurt manufacturing^51^. Thus, this finding suggested that our BLG-free milk might have stronger antimicrobial activity than that of common milk. In conclusion, we produced the hypoallergenic milk using the gene-editing technology. Our study provides an important basis for removal of more allergenic proteins in milk to produce non-allergenic “humanized milk” in future.

Applications of engineered nucleases in research, biotechnology, and medicine are hampered by their off-target effects. Both ZFNs, TALEN and Cas9 can induce mutations at off-target sites that are highly homologous to on-target sites. In our study, we using the genome sequencing to detect the potential off-target in the *BLG* bi-allelic knockout cow and found that our animal is free of off-target events. This may be suggested the high specificity of ZFNs mRNA particularly for this locus, but not all the other locus. With the technology development of reduce off-target, it may improve the application of the gene-editing farm animals^52,53^.

The germline transmission of transgenic mutations is an important concern. In this study, we proved the germline transmission of *BLG* mutations and accelerated the production of the number of offspring from the genome-editing F_0_ cows using MOET^33^. Our methods may accelerate the application of the highly valued genome-editing bovine in future. Importantly, our DNA-free gene-editing cow contains small deletions (indels) at chromosomal target sites that are indistinguishable from naturally occurring genetic variation, moreover the BLG-free milk is lower allergenicity than the ordinary milk. So, it might be exempt from current GMO regulations in future.

However, only one BLG bi-allelic knockout cow was obtained in this study, which might limit the application of large-scale production of designed milk. This finding might be primarily caused by the low efficiency of the SCNT technology^54^, Moreover, this finding is associated with increased rates of abortion and health problems due to incomplete epigenetic reprogramming of the somatic donor nuclei^20,55^. In this study, only thirty reconstructed embryos were transferred to the sixteen recipient animals. Therefore, to obtain BLG bi-allelic knockout cows, transplanting many reconstructed embryos to many recipient animals is necessary. Indeed, in our previous study^9,56,57^, more transgenic cloned animals were produced using more embryos and recipient animals. Additionally, the development of SCNT technology^58,59^ and the improvement of SCNT efficiency^60^ may accelerate the application of large-scale production of designed milk.

In summary, we generated DNA-free BLG bi-allelic knockout cow by ZFNs mRNA, which produced the hypoallergenic milk. ZFNs and other genome-editing methods can establish alternatives to transgenic (genetic modification)-based methods for the genetic improvement of livestock and the production of “designer” milk for human health benefits.

## Materials and Methods

### Ethics statement

All animal experiments including the transgenic cows experiment and Balb/c mice food allergy model were conducted in accordance with the Guide for the Animal Experimental Welfare and Ethical and were approved by the Animal Experimental Welfare and Ethical Inspection Committee in China Agricultural University (Permit Number: SKLAB-2013-06-01 for the transgenic cows’ experiment and FSNE-2017-02-05). All efforts were directed during the animal experiments to minimize suffering. This study was approved by the Institutional Review Boards of China Agricultural University, and all methods were performed in accordance with the relevant guidelines and regulations. The parents or legal guardians of all donors were provided informed consents for the study, which was approved by the ethics committee of China Agricultural University (No. 2017-04-11).

### Generation of ZFNs mRNA

The method was from our previous study^20^. Briefly, Xba I (New England Biolabs, USA) linearized the ZFNs plasmid DNAs, which included the T7 promoter, downstream of the ZFNs coding region as template (1 μg) for *in vitro* transcription (IVT). A mMESSAGE mMACHINE® T7 mRNA transcription kit (Ambion) was used to perform IVT, and the mRNA PolyA Tailing Reaction used a Poly(A) Tailing Kit (Ambion), followed by purification of the tailed mRNA with a MEGAClearTM kit (Ambion). The mRNAs were diluted to approximately 500 ng/μL for transfection and stored at −80 °C.

### Transfection of primary fibroblasts

The primary female bovine fetus fibroblasts (BFFs) was transfected with the BLG-ZFNs mRNA using program T-016 on a Nucleofector^TM^ (AMAXA) from our previous method^20^. A co-transfection ratio of 1:1 was used and the total mRNA input was 4 μg/10^6^ cells. Limiting dilution was used to isolate cell colonies forming 24-48 h after transfection, and the cell concentration was approximately 500 cells/dish (10 cm^2^). Individual cell clones were isolated 7-10 days after dilution of the culture, after which the culture was expanded and their sequences were analyzed. Cells were cryopreserved after a total of 12-14 days in culture.

### Identification of the *BLG*-targeted positive clone

Genomic DNA was extracted from single cell colonies using a DNeasy Blood and Tissue kit (Qiagen, Hilden, Germany). The primers included the Forward BLG primer: 5′AGGCCTCCTATTGTCCTCGT3′ and the Reverse BLG primer: 5′GCAAAGGACACAGGGAGAAG3′. Amplification conditions were as follows: 94 °C for 10 min, then 35 cycles of 94 °C for 1 min, 54 °C for 30 s and 72 °C for 30 s, followed by extension at 72 °C for 10 min. The PCR products were TA cloned and sequenced.

### Somatic cell nuclear transfer

The cloned cows were all generated by somatic nuclear transfer. The procedures for *in vitro* maturation (IVM) of bovine oocytes were described previously^20^. Briefly, cumulus-oocyte complexes (COCs) were aspirated from follicles and matured for 18-20 h in maturation medium (TCM199, 10% fetal bovine serum (FBS), 0.01 U/ml bovine follicle-stimulating hormone (bFSH), 0.01 U/ml bovine luteinizing hormone (bLH), 1 mg/ml estradiol-17) (Life Technologies). Mature oocytes were enucleated and fused with the donor cells enriched in G_0_ of the cell cycle with two DC pulses of 2.5 kV/cm for 10 ms each at 1 s apart, delivered using a BTX2001 Electro Cell Manipulator (BTX, San Diego, CA, USA). The reconstructed embryos were activated in medium supplemented with 10 mg/ml cycloheximide and 2.5 mg/ml cytochalasin D and were cultured to form blastocysts at day 7. The high-quality blastocysts were transferred into synchronized recipient cows 7 days after estrus (two blastocysts/recipient). Pregnant cows were monitored by rectal palpation at regular intervals.

### Identification of the *BLG*-targeted cow

Genomic DNA was extracted from the ears of newborn calve by a DNeasy Blood and Tissue kit (Qiagen, Hilden, Germany). Subsequently, the genomic DNA was examined via PCR. The primers included the Forward BLG primer: 5′AGGCCTCCTATTGTCCTCGT3′ and the Reverse BLG primer: 5′GCAAAGGACACAGGGAGAAG3′. Amplification conditions were as follows: 94 °C for 10 min, then 35 cycles of 94 °C for 1 min, 54 °C for 30 s and 72 °C for 30 s, followed by extension at 72 °C for 10 min. The expected amplification length was 546 bp. The PCR products were TA cloned and sequenced.

### Collection of transgenic milk

Natural lactation milk samples were collected on days 1, 3, 5, 7, 9, 11, 13, 15, 17, and 19 from the BLG-targeted #111027 cow and wild-type cow. Twenty-five liters of milk were obtained on an average day, and part of the collected milk was centrifuged at 12,000 g for 5 min to defat, and the total protein concentration was estimated for further analysis. One hundred-liter samples of *BLG*-free milk and control milk were freeze-dried to prepare milk powder of which part was used to perform the allergenicity assay.

### SDS-PAGE, Western blotting and ELISA analyses

Milk samples from the BLG-targeted cow and wild-type cows were collected. A 1.0 mL milk protein sample was centrifuged at 12,000 g for 5 min to defat, and then the defatted milk samples containing 30 µg of milk protein as estimated by the method of BCA (Beyotime, Shanghai, China) were analyzed by SDS-PAGE and Western blot analysis. For SDS-PAGE, milk protein samples were separated on 15% tris-glycine polyacrylamide gels under denaturing and reducing conditions, and the protein contents were quantified by dying the gels with Coomassie brilliant blue. For Western blotting analysis, the diluted milk samples were separated on 15% tris-glycine polyacrylamide gels under denaturing and reducing conditions and were then transferred to polyvinyl difluoride membranes (Invitrogen Corporation, Carlsbad, CA, USA), which were incubated with a polyclonal anti-bovine BLG antibody (dilution, 1:10000; United States Biological, Inc., Swampscott, MA, USA) and a horseradish peroxidase (HRP)-conjugated secondary anti-rabbit IgG antibody (dilution, 1:10000; Sino-American Co., Beijing, China). We used BCA to detect the total protein concentration and the commercial ELISA kits (eBioscience, Inc., San Diego, CA) for the main proteins (BLG, casein, α-whey protein, BSA, lactoferrin and immunoglobulin) in the milk.

### Establishment of food allergy model in Balb/c mice

Balb/c mice in our research were obtained from the Vital River Laboratories, Inc. (Beijing, China). The study was conducted in the specific pathogen free (SPF) animal laboratory of College of Food Science and Nutritional Engineering, China Agricultural University (Beijing, China). Animal rooms were maintained with temperature of 22 ± 1 °C, humidity of 55 ± 5%, a 12 h light/dark cycles and air exchanges at 15 times/h. Feed and water were supplied ad libitum. The commercial SPF rodent maintenance feed produced by Ke Ao Xie Li feed Co. Ltd. (Beijing, China) met the Chinese Standard GB14924.3-2010. Animal experiments in our research were conducted in accordance with the Guide for the Animal Experimental Welfare and Ethical in the Food Science and Nutritional Engineering College of China Agricultural University and were approved by the Animal Experimental Welfare and Ethical Inspection Committee in China Agricultural University. All efforts were directed during the animal experiments to minimize suffering. Ninety-six female Balb/c mice (5-6 weeks old) were randomly divided into 8 groups having an initial body weight difference of ± 20%: Negative control, Positive control, Control milk powder (1 mg, 2 mg or 4 mg total protein in the control milk powder samples) and BLG-free milk (1 mg, 2 mg or 4 mg total protein in the BLG-free milk powder samples). Sensitization was achieved in all groups by oral administration of different dose proteins (OVA in the positive control, control milk and BLG-free milk) in 200 μL PBS on days 0, 7, 14, 21 and 28. Mice in the Control group were only gavaged with PBS at the sensitization time. All the mice were subsequently challenged with 10-fold protein dose of milk powder samples (the negative and positive control groups were all challenged with OVA) on day 42 (Fig. 3A).

### Body weights and the rectal temperature drop

Body weight was measured and recorded weekly and rectal temperature was measured before and 40 min after the challenge using a WI88375 probe (Beijing Science and Technology, Beijing, China).

### Allergen-specific IgE level in serum

Blood samples were collected from the angular vein plexus of the animals in every group at 30 min after challenge on day 42. The serum was stored at −20 °C to measure allergen-specific IgE using an ELISA method, according to an established procedure^28,29^. The specific antibody determination was as follows: 96-well plates were coated with allergen (OVA, control milk or BLG-free milk, 2 μg/well) dissolved in carbonate bicarbonate buffer (pH 9.6) and incubated over night at 4 °C. Then, after washing 3 times with PBST (0.05% Tween 20/PBS), 200 μL of BSA solution was added to the wells (1% BSA/PBS) and incubated for 1 h at 37 °C. mouse serum (the dilution of 1:10) were added to the wells (100 μL/well) after washing 3 times. The plates were again incubated for 1 h at 37 °C and washed 3 times. Next,100 μL/well of a diluted HRP-conjugated Goat anti-Mouse IgE (Abcam) (2000 × diluted) was added and incubated for 1 h at 37 °C. After washing 6 times, 100 μL of TMB was added and incubated in the dark for 15 min at 37 °C. Then, 50 μL of H_2_SO_4_ (2 M) was added to the wells and the OD at 450 nm was read using a Multiskan MK3 automated microplate reader (Thermo Scientific).

### Pepsin digestion assay

Pepsin digestion assay of protein samples was performed according to the No. 869 bulletin of the Ministry of Agriculture food safety detection of genetically modified organisms and derived products: Method of target protein digestive stability in simulative gastric and intestinal fluid^61^. Control and transgenic milk powders were subjected to *in vitro* gastric digestion at a 5 mg/mL final concentration. In brief, the proteins were dissolved in simulated gastric fluid (SGF, 2 g/L NaCl) at pH 1.2, preheated for 5 min at 37 °C and subjected to an *in vitro* gastric digestion with porcine pepsin (3200-4500 U/mg protein, Sigma-Aldrich) at an enzyme/substrate ratio of 19:1. At each time point (0 s, 15 s, 2 min, 30 min and 60 min), 200 µL of the reaction mixture was transferred to a sampling tube that contained 70 µL of 5 × Laemmli buffer (40% glycerol, 5% 2-mercaptoethanol, 10% SDS, 0.33 M Tris, 0.05% bromophenol blue, pH 6.8) and 70 µL of 800 mM NaHCO_3_. All neutralized samples were then boiled at 100 °C for 5 min and subjected to SDS-PAGE.

### Human serum IgE binding analysis by ELISA and Western blotting

We collected each 5 mL sera from six verified CMA children and five non-allergic volunteers recruited from the Peking University Third Hospital (Beijing, China). This study was approved by the Institutional Review Boards of China Agricultural University, and all donors provided informed consent. For ELISA, the method was described in the Allergen-specific IgE level in serum. The plates were incubated with 100 μL of biotin-conjugated polyclonal anti-human IgE diluted 1:1000 in antibody dilution medium for 1 h at 37 °C followed by 100 μL of HRP-conjugated rabbit anti-biotin diluted at 1:2000 in an antibody dilution for 1 h at 37 °C. For Western blotting, control and BLG-free milk powders were separated by SDS-PAGE, electroblotted onto a nitrocellulose membrane and incubated for 1 h at RT with 5% BSA in TBST. The membrane was incubated overnight at 4 °C with dilutions of the sera from allergy patients. Bound IgE antibodies were detected using HRP-conjugated mouse anti-human IgE diluted 1:1000 in TBST. The strips were light-resistant and were incubated with TMB substrate at RT for 10 min. The reaction was stopped with distilled water. After termination of the reaction, the membrane was exposed to film.

### Off-Target

DNA from DNA-free *BLG* bi-allelic knockout cow #111027 was submitted to the BGI-Shenzhen company and prepared for Illumina sequencing library construction. 19X sequence data were generated on HiSeq platform with PE125 mode. Sequencing data of the two samples were mapped onto the bovine reference genome (UMD3.1.1) with BWA (Version 0.7.10-r789), and Indels were called by GATK (Version 3.5-0-g36282e4). We checked the ZFNs-specific indels to a close proximity of those potential off-target sites by custom Perl script. Briefly, the spacer between ZFNs arms was allowed to be 4–16 bp, mismatches of each ZFNs monomer (17 bp) were up to 5 bp. For all matching sequences computed, we extract their corresponding information for comparison with de novo indels of #111027. BEDTools was adopted to find de novo indels within 20 bp distance of predicted potential targets for the edited animal.

### MOET assay

The procedures for MOET were described previously^33^. Briefly, in the morning of the first day of the estrus cycle, the CUE-Mate was injected on the 0th day. From the 5th to the 8th day, Folltropin-V was injected twice daily at 8:00 am and 8:00 pm, and the concentrations were 24.0 mg, 18.0 mg, 12.0 mg, and 6.0 mg, respectively. Propylene glycol (PG), 4.0 mL, was injected on the 7th day after injection of Folltropin-V and after withdrawal of the plug during the morning injection of Folltropin-V on the 8th day. The early estrus was observed on the 9th day. The first breeding was performed 12 h after estrus. The second breeding was performed at intervals of 12 h. The non-surgical donor embryos were flushed on the 16th day and transplanted into recipient cows on the 7th day after estrus.

### Statistical analysis

Statistical analysis of data was performed using GraphPad Prism version 5.0 for Windows (Inc.7825 Fay Avenue, Suite 230 La Jolla, CA 92037 USA). One-way ANOVA followed by Tukey’s multiple comparison test was used for comparisons between groups with normal distributions. Differences between experimental groups were regarded as significant when P ≤ 0.05.

## Electronic flementary material

Supplementary Information
