# Supplementary material for: Production of hypoallergenic milk from DNA-free beta-lactoglobulin (BLG) gene knockout cow using zinc-finger nucleases mRNA
Source: Sci Rep. 2018 Oct 18;8:15430. doi: 10.1038/s41598-018-32024-x (PMC6194018; doi:10.1038/s41598-018-32024-x)
Supplement: Supplementary file 1 — Supplementary Information [file 41598_2018_32024_MOESM1_ESM.docx]

**SUPPLEMENTARY INFORMATION**

**Production of hypoallergenic milk from DNA-free beta-lactoglobulin (*BLG*) gene knockout cow using zinc-finger nucleases mRNA**

# Zhaolin Sun1, †, Ming Wang1, †, Shiwen Han2, †, Zhiyuan Zou1, Fangrong Ding1, Xinrui Li2,

Ling Li1, Bo Tang3, Haiping Wang1, Ning Li1, Huilian Che2*, Yunping Dai1*

1. State Key Laboratory for Agrobiotechnology, College of Biological Sciences, China Agricultural University, Beijing, China
2. Beijing Advanced Innovation Center for Food Nutrition and Human Health, College of Food Science

& Nutritional Engineering, China Agricultural University, Beijing, China

1. Beijing Genprotein Biotechnology Company, Beijing, China

† These authors contributed equally to this work

*. Correspondence and requests for materials should be addressed to H.C. Email: [chehuilian@cau.edu.cn](mailto:chehuilian@cau.edu.cn) or Y.D. Email: [YDai@cau.edu.cn](mailto:YDai@cau.edu.cn)

Address: Beijing city, Haidian district, Yuanmingyuan west road #2, China Agricultural University. Telephone number: +86 13621040689


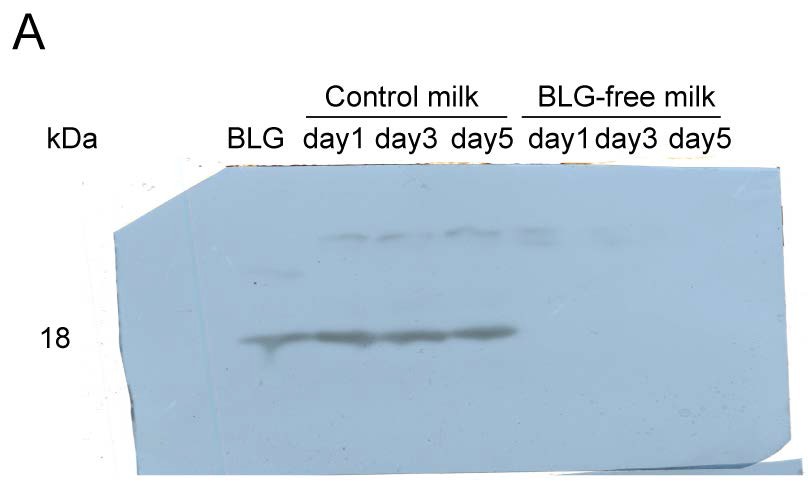


**Supplementary FIG. S1.** Full-length blots of Fig.2 B.

# **The whole image of the Western blot.** A Characterization of the BLG-free milk by Western blot. BLG, 2.5 µg of commercial BLG (Sigma) as a positive control; line 2-4, 1 µl of wild-type cow raw milk from different days; line 5-7, 1 µl of #111027 (BLG-free) cow raw milk from different days.

Control milk powder BLG-free milk powder

18 kDa


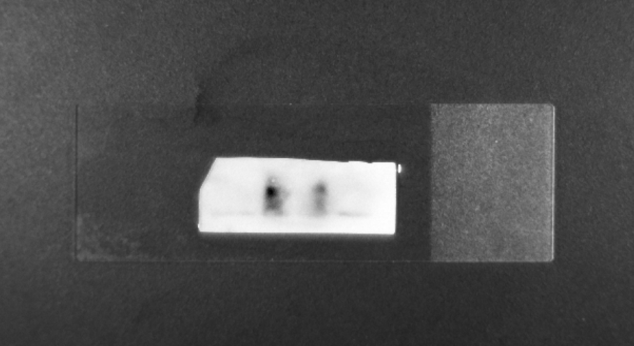


**Supplementary Fig. S2** Full-length blots of Fig.4 C.

# **The whole image of the Western blot.** All of the blots were the first exposure in this Fig. and the NC membranes were tailored according to the suitable protein molecule weight （from 10 kDa to 25 kDa）prior to incubating with human serum.
